# Ultraprocessed foods consumption and risk of preeclampsia: a secondary analysis of the improving mothers for a better prenatal care trial Barcelona (IMPACT BCN) randomized clinical trial

**DOI:** 10.1016/j.ajcnut.2026.101255

**Published:** 2026-03-02

**Authors:** Alejandra Trejo-Domínguez, Leticia Benitez, Francesca Crovetto, Rosa Casas, Lina Youssef, Marta Larroya, Ana María Ruiz-León, Ayako Nakaki, Mariona Genero, Irene Casas, Rommy H Novoa, Noelia Encabo, Michael Rahman, Xiaosuo Wang, John F O’Sullivan, Narelle R Hegarty, Eduard Vieta, Eduard Gratacós, Ramon Estruch, Fàtima Crispi, Sara Castro-Barquero

**Affiliations:** 1 BCNatal, Barcelona Center for Maternal and Fetal Medicine (Hospital Clínic and Hospital Sant Joan de Déu), University of Barcelona, Barcelona, Spain; 2 Institut de Recerca Sant Joan de Déu, Esplugues de Llobregat, Barcelona, Spain; 3 Institut d’Investigacions Biomèdiques August Pi i Sunyer (IDIBAPS), Barcelona, Spain; 4 Spanish Network in Maternal, Neonatal, Child and Developmental Health Research, RICORS-SAMID, RD24/0013/0004, Instituto de Salud Carlos III, Madrid, Spain; 5 Departament de Ciències Clíniques, Facultat de Medicina i Ciències de la Salut, Universitat de Barcelona, Barcelona, Spain; 6 Centro de Investigación Biomédica en Red de Fisiopatología de la Obesidad y Nutrición (CIBEROBN), Madrid, Spain; 7 Institut de Recerca en Nutrició i Seguretat Alimentària (INSA-UB), University of Barcelona, Barcelona, Spain; 8 Department of Internal Medicine Hospital Clinic, University of Barcelona, Barcelona, Spain; 9 Dexeus Mujer (Hospital Universitari Dexeus), Barcelona, Spain; 10 Charles Perkins Centre, School of Medical Sciences, Faculty of Medicine and Health, The University of Sydney, Sydney, New South Wales, Australia; 11 CardioMetabolic Medicine Laboratory, The University of Sydney, Camperdown, New South Wales, Australia; 12 School of Medical Sciences, Faculty of Medicine and Health, The University of Sydney, Camperdown, New South Wales, Australia; 13 Department of Cardiology, Royal Prince Alfred Hospital, Camperdown, New South Wales, Australia; 14 Charles Perkins Centre, The University of Sydney, Camperdown, New South Wales, Australia; 15 Department of Medicine, TU Dresden, Helmholtzstr, Dresden, Germany; 16 ANSTO, Lucas Heights, New South Wales, Australia; 17 Department of Psychiatry and Psychology, Hospital Clinic, Neuroscience Institute, University of Barcelona, IDIBAPS, Center for Biomedical Research on Mental Health (CIBERSAM), Barcelona, Spain; 18 Centre for Biomedical Research on Rare Diseases (CIBERER), Barcelona, Spain

**Keywords:** preeclampsia, ultraprocessed foods, pregnancy, prenatal, Mediterranean diet

## Abstract

**Background:**

Preeclampsia is a pregnancy-specific condition affecting 2%–8% of pregnant females and a leading cause of maternal and perinatal morbimortality. High ultraprocessed food (UPF) consumption has been associated with the development of noncommunicable chronic diseases, but evidence on pregnancy outcomes is scarce.

**Objectives:**

To study the association of maternal UPF consumption and the risk of preeclampsia.

**Methods:**

This study is a secondary analysis of the IMPACT BCN, a randomized clinical trial including 1221 pregnant females at high risk for small for gestational age newborns, conducted in Barcelona, Spain. Among these, 812 participants with complete dietary data at 2 timepoints during pregnancy (between weeks 19–23 of gestation and weeks 34–36 of gestation) were included in this analysis and classified into tertiles of change in UPF consumption during pregnancy. Dietary UPF consumption was assessed using NOVA classification with a validated 151-item food frequency questionnaire. Preeclampsia was defined as high blood pressure plus targeted organ involvement. Analyses were performed using logistic regression models adjusted for potential confounding factors.

**Results:**

Associations between change of UFP consumption during pregnancy and overall preeclampsia were observed across tertiles [odds ratio (OR)_T3 compared with T1_: 2.29; 95% confidence interval (CI): 1.06, 4.97, *P*-trend 0.026], but not when UPF change was modeled as a continuous variable [OR: 1.04; 95% CI: 0.95, 1.14]. Among UPF subclasses, pre-prepared dishes were significantly associated with preeclampsia risk [OR_T3 compared with T1_: 2.36; 95% CI: 1.09, 5.12].

**Conclusions:**

In a high-risk population, a higher change in dietary intake of UPF consumption from the second to third trimester of pregnancy was associated with a higher risk of preeclampsia.

This trial was registered at clinicaltrials.gov as NCT03166332.

## Introduction

Preeclampsia is a pregnancy-specific condition affecting 2%–8% of pregnancies, and is a leading cause of maternal and perinatal mortality and morbidity [1]. It is also linked with long-term maternal health risk, such as an increased cardiometabolic risk [[2], [3], [4]]. Although its exact cause remains unclear, traditional theories suggest that disturbances in placentation lead to maternal endothelial damage and increased cardiovascular risk, with lifestyle factors playing substantial roles [[5], [6], [7], [8], [9]]. Recent studies highlight maternal diet as a key factor influencing the development of preeclampsia and adverse perinatal outcomes [10]. A nutrient-dense diet is essential during pregnancy, as Western dietary patterns linked to poor diet quality can increase the risk of excessive weight gain, maternal obesity, and gestational diabetes [[11], [12]].

Consumption of ultraprocessed foods (UPFs), a marker of poor diet, is rising rapidly, especially in middle and high-income countries [13]. The NOVA classification groups food based on processing levels, with UPF being the most processed and least nutritious [14]. In nonpregnant population, high UPF consumption is associated with chronic diseases, such as cardiovascular disease [15,16], hypertension [17], and obesity [18,19]. During pregnancy, increased UPF consumption correlates with a higher risk of preeclampsia, especially in females with higher prepregnancy BMI and maternal age (≥30 y) [10]. A meta-analysis reported a 28% increased likelihood of developing preeclampsia because of high UPF consumption, although, as all studies included were observational, causality cannot be established. Moreover, inconsistencies in dietary assessment methods and UPF classification pose challenges in accurately determining the strength of this association [20]. In this context, the incorporation of objective biomarkers, such as nitrogen and carbon isotope ratios, which have been associated with certain ingredients present in UPF, can help improve the accuracy of dietary assessment. Although these findings highlight the potential role of maternal diet in preeclampsia, further prospective studies with standardized dietary assessments and robust control for confounding factors are necessary to confirm these associations.

This study aimed to evaluate the association between changes in maternal UPF consumption and the risk of preeclampsia using available data from the Improving Mothers for a better PrenAtal Care Trial BarCeloNa (IMPACT BCN) trial. We hypothesized that a greater consumption of UPF during pregnancy would be associated with an increased risk of preeclampsia among high-risk pregnant individuals. We also aimed to assess the effect of maternal lifestyle interventions—promotion of Mediterranean diet and stress reduction—during pregnancy on dietary consumption of UPF.

## Methods

### Study population and design

This study is a secondary analysis of the IMPACT BCN trial, a randomized clinical trial with parallel groups conducted at a BCNatal (Hospital Clinic and Hospital Sant Joan de Déu), a major reference center for maternal-fetal and neonatal medicine in Barcelona, Spain (2017–2020) including 1221 pregnancies at high risk for small for gestational age newborns, randomly allocated at 19–23 wk gestation into 3 groups: *1*) a Mediterranean diet intervention, *2*) a stress reduction program based on mindfulness techniques, or *3*) usual care. Details of the study protocol and the primary outcome of the IMPACT BCN trial have been described elsewhere [21,22]. The Institutional Review Board from Hospital Clinic approved the study (HCB-2016-0830); all pregnant females provided written informed consent, and the trial was registered in clinicaltrials.gov Identifier (NCT03166332).

For this analysis, from the initial 1221 sample size, some participants were excluded: 322 for missing data on dietary information at baseline and/or final visit, 50 for energy intake outside predefined limits [23, 27[23], 27 withdrew consent, and 10 because of fetal/neonatal malformations, ending with a final sample size of 812 participants included in the analysis. Given a baseline preeclampsia prevalence of 6.6% in our cohort and ∼270 participants per tertile, the detectable difference between groups at 80% power and α = 0.05 would correspond to an absolute difference of ∼6% in outcome frequency, consistent with the original sample size assumptions for the trial (Supplementary Figure 1)**.**

### Assessment of maternal diet during pregnancy

Maternal diet during pregnancy was assessed using a 151-item semiquantitative food frequency questionnaire (FFQ) validated for the present study population [24], and a validated Mediterranean diet assessment score (preg-MEDAS) [25]. Both questionnaires were administered by trained dietitians in face-to-face interviews in all participants during enrollment (19–23 wk gestation) and the final visit (34–36 wk gestation). The FFQ at enrollment assessed dietary intake from onset of pregnancy, whereas the third-trimester FFQ captured intake from previous visit. Food consumption derived from the FFQ was converted into energy and nutrient intake with the Centre d'Ensenyament de Nutrició Humana i Dietètica (CESNID) and Moreiras composition tables using traditional recipes [26,27]. Details of the FFQ validations were described elsewhere [24]. Briefly, participants indicated their usual and frequent consumption of listed food items in the FFQ, based on 9 frequency categories (ranging from never or <1 time/mo to ≥6 times/d) and using common units or portion sizes. A total of 15 food groups were listed: milk and dairy products, cereals and whole grains, vegetables, legumes, sausages, oils and fats, eggs, meat and fish, fast food, canned products, fruit, nuts, sweets and desserts and others (salt and sugar), and alcoholic and nonalcoholic beverages.

### Maternal dietary UPF consumption

For the estimation of UPF consumption, items in the FFQ were classified according to the NOVA system [13,28,29], developed by the Public Health Faculty of the University of São Paulo in Brazil. This system classifies foods and beverages according to the nature, extent, and purpose of their industrial processing into 4 groups: *1*) unprocessed or minimally processed foods (i.e., fresh or frozen fruits and vegetables, eggs, pasteurized milk, meat, seeds, nuts, grains, or plain yogurt); *2*) processed culinary ingredients (i.e., oils, fats, sugar, and salt); *3*) processed foods (i.e., canned vegetables, canned fish, fruits in syrup, cheeses, beer, and wine); and *4*) UPF (i.e., soft drinks, sweet, or savory packed snacks, processed meats, pre-prepared frozen dishes, and “instant” products). Two independent dietitians oversaw the classification procedure, whereas inconsistencies in classifying specific food items were resolved through extensive deliberations with researchers’ experts in nutrition. Details about the allocation of FFQ items to processing groups with examples are provided in Supplementary Table 1. Furthermore, items belonging to the UPF group (foods and beverages) were allocated into the following subgroups: dairy products; processed meat; pre-prepared dishes; sweets; and nonalcoholic beverages (Supplementary Table 2).

### Definition of preeclampsia

Preeclampsia was defined according to current guidelines [30,31], as systolic blood pressure ≥140 mm Hg or diastolic blood pressure ≥90 mm Hg ≥4 h apart after 20 wk of gestation plus proteinuria of ≥300 mg in 24 h or other maternal organ dysfunction including increased creatinine, elevated transaminases, right upper or epigastric pain, neurological complications (eclampsia, altered mental status, blindness, stroke, clonus, severe headache, persistent visual scotomata), thrombocytopenia, hemolysis, disseminated intravascular coagulation or uteroplacental dysfunction (fetal growth restriction, abnormal umbilical artery Doppler or stillbirth). The occurrence of preeclampsia was collected from medical records by obstetricians. Cases of preeclampsia were subdivided into early (delivery <34 wk) and late onset (delivery ≥34 wk) [32].

### Assessment of the molecular carbon and nitrogen isotope ratio of maternal plasma

As part of the study protocol, peripheral blood samples were collected from participants at the end of pregnancy. In a random subsample of 532 participants, carbon δ^13^C (^13^C/^12^C) and nitrogen δ^15^N (^15^N/^14^N) isotope ratios were performed to assess molecular enrichment of higher isotope ratio, known to be related to UPF consumption [33]. Values were reported in parts per thousand (‰ or per mil) relative to international standards. Plasma samples (3 μL) were transferred to a tin cup, dried at 50°C for ∼3 h, weighed and analyzed via an elemental analyzer (Thermo Fisher Scientific). The combusted gases (N_2_ and CO_2_) were analyzed using a CF-IRMS (Thermo Fisher Scientific Conflo IV, Delta V Advantage IRMS). Isotope data were calibrated using International Atomic Energy Agency secondary standards certified relative to VPDB for carbon and AIR for nitrogen. The method achieved an accuracy of ±1% for N% and C% and ±0.3‰ for δ^15^N and δ^13^C. Two quality-control reference samples were included in each run and 10% of samples were analyzed in duplicate. The limits of detection for N% and C% were determined according to daily linearity tests.

### Covariates

Maternal characteristics were obtained from questionnaires and dietary questionnaires were administered by trained dietitians in a face-to-face interview at trial enrollment (19–23 wk gestations) and final visit (34–36 wk gestation) [21]. Data collected included maternal age, ethnicity, socioeconomic status (low/medium/high; defined as low if participants reported having never worked or being unemployed for >2 y and having a partner with unqualified work or who was unemployed; high if they reported university studies regardless of whether they were working; and medium if any other situations), educational level (primary school/secondary school/university; defined as the highest educational degree achieved), self-reported prepregnancy BMI as kg/m^2^, chronic hypertension, diabetes, parity (multiparous/nulliparous), adverse obstetrical history (previous fetal growth restriction, preeclampsia, or stillbirth), use of assisted reproductive technologies, and smoking during pregnancy. Obesity was defined if prepregnancy BMI was ≥30 kg/m^2^. Occurrence of small for gestational age newborns (defined as birthweight below the 10th centile), severe small for gestational age (birthweight below the 3rd centile) [34,35], preterm birth (delivery before 37 wk of gestation) and adverse perinatal outcomes (≥1 of the following: preterm birth, preeclampsia, perinatal mortality, severe small for gestational age, neonatal acidosis, low Apgar score, or presence of any major neonatal morbidity) were also evaluated.

### Statistical analysis

Categorical variables were described as frequencies, whereas the continuous variables were expressed as means and SD. According to the trial’s statistical analysis plan, no imputations of missing data were performed for the analyses of the secondary end points or variables, and this study was conducted with participants with available data [21]. The association between maternal UPF consumption and preeclampsia in the whole study population was assessed by comparing tertiles of change in dietary UPF consumption (baseline compared with final visit) using the lowest tertile (reduction in UPF consumption) as the reference category, and as a continuous variable (per 50 g of change). Data were expressed using odds ratio (OR) and their corresponding 95% confidence interval (CI). ORs were estimated by logistic regression models: *1*) Model 1 was controlled for age (<40; ≥40), socioeconomic status (low compared with high), baseline energy intake (kcal/d), ethnicity (White compared with not White), prepregnancy BMI (<30/≥30 kg/m^2^), and intervention arm; *2*) Model 2 was further adjusted by nulliparity (yes/no), smoking during pregnancy (yes/no), previous hypertensive disorder (yes/no), and the use of assisted reproductive technologies (yes/no). To assess the linear trend (*P*-trend) across tertiles of UPF, the mean value was assigned to each tertile.

Changes in overall UPF consumption and by individual UPF subclasses during the intervention were assessed by analysis of covariance adjusted for the baseline levels of each variable. Within- and between-group differences were expressed as estimated means and 95% CI. To account for multiple testing, *P* values were adjusted using the Benjamini–Hochberg false discovery rate (FDR) procedure. An FDR-adjusted *P* value ≤ 0.05 was considered statistically significant. The associations between UPF consumption and isotopic nitrogen and carbon biomarkers were analyzed using 1-way analysis of variance. Bonferroni correction was applied for multiple comparisons to assess differences between tertiles of change (baseline compared with final visit, using the lowest tertile as the reference category) of UPF and UPF subclass consumption. The results are expressed as mean and SD. All analyses were performed using Stata (16.0, StataCorp LP) and Statistical Package for the Social Sciences Statistical Software Package version 27.0 (SPSS Inc). *P* value <0.05 was considered statistically significant.

## Results

### Characteristics of the study population according to UPF consumption

Baseline characteristics of the study population are shown in Table 1, distributed by tertiles of change in UPF consumption. On the basis of maternal measurements, females classified in the highest tertile of UPF consumption show higher preconceptional body weight, BMI, and educational level. Meanwhile, ethnicity distribution varied across UPF tertiles, a higher proportion of White participants were in tertile 3. The remaining maternal characteristics were similar among UPF groups. Details about baseline characteristics according to initial consumption of UPF are available in Supplementary Table 3.

**TABLE 1:** Characteristics of the study population according to changes in the consumption of ultraprocessed foods (UPFs)

	All women	Changes in UPF consumption (final vs. baseline)		
		UPF tertile 1 (−1342 to −57.2 g/d)	UPF tertile 2 (−57.2 to 10.5 g/d)	UPF tertile 3 (10.5 to 1453 g/d)
*n* (%)	812 (100)	271 (33)	271 (33)	270 (33)
Baseline UPF consumption	225.9 (151.1)	321.9 (187.7)	183.8 (103.4)	171.9 (93.2)
Age (y)	37.3 (4.6)	37.1 (4.7)	37.5 (4.3)	37.2 (4.8)
Ethnicity				
White	673 (82.9)	210 (77.5)	240 (88.6)	223 (82.6)
Latin	103 (12.7)	43 (15.9)	24 (8.9)	36 (13.3)
Asian	12 (1.5)	6 (2.2)	4 (1.5)	2 (0.7)
Afro-American	10 (1.2)	6 (2.2)	0 (0)	4 (1.5)
Others	14 (1.7)	6 (2.2)	3 (1.1)	5 (1.8)
Smoking habit				
No	658 (81.0)	219 (80.8)	220 (81.2)	219 (81.1)
Stop during pregnancy	102 (12.6)	33 (12.2)	31 (11.4)	38 (14.1)
Yes	52 (6.4)	19 (7.0)	20 (7.4)	13 (4.8)
Educational level				
Primary school	33 (4.1)	11 (4.1)	9 (3.3)	13 (4.8)
Secondary school	157 (19.3)	66 (24.3)	35 (12.9)	56 (20.7)
University	622 (76.6)	194 (71.6)	227 (83.8)	201 (74.4)
Employment status				
Student	7 (0.9)	2 (0.7)	1 (0.4)	4 (1.5)
Employed	669 (82.4)	222 (81.9)	225 (83.0)	222 (82.2)
Autonomous	60 (7.4)	13 (4.8)	24 (8.9)	23 (8.5)
Housekeeper	23 (2.8)	11 (4.1)	6 (2.2)	6 (2.2)
Unemployed	53 (6.5)	23 (8.5)	15 (5.5)	15 (5.6)
Nulliparity	477 (58.7)	111 (41.0)	111 (41.0)	113 (41.8)
Use of assisted reproductive technologies	217 (26.7)	69 (25.5)	77 (28.4)	71 (26.3)
Previous hypertensive disorder	31 (3.8)	9 (3.3)	11 (4.1)	11 (4.1)
Weight (kg)				
Preconceptional	63.5 (12.8)	63.1 (12.3)	62.2 (11.6)	65.3 (14.2)
19–23 wk gestation	69.5 (12.2)	69.3 (11.8)	68.2 (11.1)	71.1 (13.5)
BMI (kg/m^2^)				
Preconceptional	23.8 (4.6)	23.8 (4.6)	23.2 (4.3)	24.2 (5.0)
19–23 wk gestation	26.0 (4.5)	26.1 (4.4)	25.6 (4.2)	26.3 (4.9)
Blood pressure (mmHg) at 19–23 wk gestation				
Systolic	105.2 (11.8)	104.0 (10.1)	105.5 (12.9)	106.2 (12.2)
Diastolic	67.6 (8.4)	66.7 (7.4)	67.9 (8.6)	68.2 (9.2)

Values are presented as the means (SD) for continuous variables and *n* (%) for categorical variables. Changes in UPF consumption was divided into tertiles of change in UPF consumption from baseline to final visit, being tertile 1 the lowest consumption of UPF.

**FIGURE 1:**
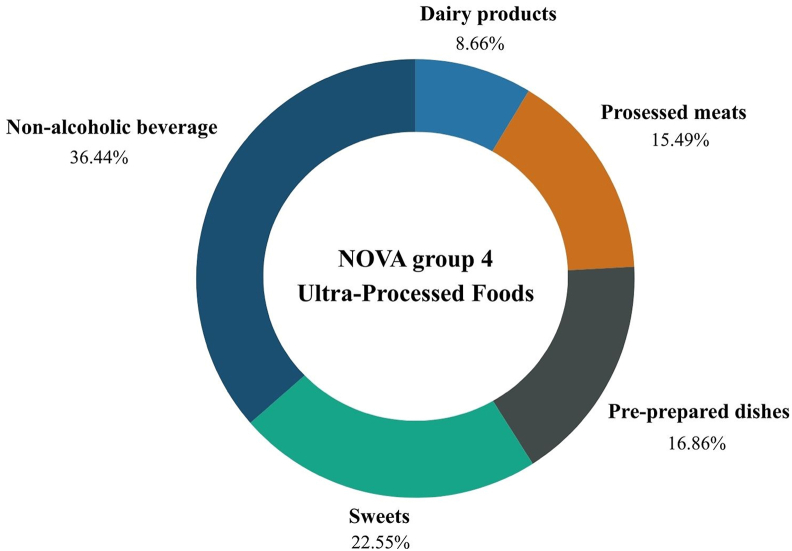
Relative contribution of different food subclasses to ultraprocessed food consumption in the overall population at baseline. Percentage of the relative intake of each food subclasses included in the NOVA 4 classification.

At baseline, the primary sources of UPF consumption were nonalcoholic beverages and sweets, 36.44% and 22.56%, respectively. At this timepoint, 16.6% of the daily energy intake was from UPF. Figure 1 details the relative contribution of all UPF subclasses to the diet.

### Changes in UPF consumption during pregnancy and risk of preeclampsia

The incidence of preeclampsia was 6.6%. When classified by the time of diagnosis, out of 54 females, 92.5% (*n* = 50) developed late-onset preeclampsia, whereas only 7.5% (*n* = 4) had an early-onset form.

Females in the highest tertile of change in UPF consumption during pregnancy had 2.31 times (95% CI: 1.06, 5.00) higher risk of developing preeclampsia compared with those in the lowest tertile. A significant positive trend was observed across tertiles, indicating that increases in UPF consumption were associated with a higher risk of preeclampsia (*P*-trend = 0.027). For late-onset preeclampsia, we noted a nonsignificant trend toward higher risk with increasing maternal UPF consumption [OR_T3 compared with T1_ of 2.14 (95% CI: 0.97, 4.71); OR_T2 compared with T1_ of 1.13 (95% CI: 0.48, 2.65)]; however, *P*-trend across tertiles was statistically significant (*P* = 0.047). Furthermore, a 50 g/d increase in consumption of UPF was not significantly associated with overall preeclampsia [Model 1: OR of 1.03 (95% CI: 0.95, 1.12); Model 2: OR of 1.04 (95% CI: 0.95, 1.14)]. Similar findings were observed for late-onset preeclampsia [Model 1: OR of 1.02 (95% CI: 0.94, 1.11); Model 2: OR of 1.03 (95% CI: 0.94, 1.13)], details shown in Table 2.

**TABLE 2:** Association between changes in total ultraprocessed food (UPF) consumption during pregnancy and the occurrence of preeclampsia

	Continuous per 50 g in UPF change	OR (95% confidence interval)			Adjusted *P*-trend
		UPF tertile 1 (−1342, −57.2 g/d)	UPF tertile 2 (−57.2, 10.5 g/d)	UPF tertile 3 (10.5, 1453 g/d)	
Overall preeclampsia					
*n* cases/*n* total: 54/812	—	12/271	15/271	27/270	—
Model 1	1.03 (0.95, 1.12)	1 (ref)	1.30 (0.58, 2.91)	2.21 (1.06, 4.63)	0.027
Model 2	1.04 (0.95, 1.14)	1 (ref)	1.17 (0.50, 2.70)	2.31 (1.06, 5.00)	0.025
Late preeclampsia					
*n* cases/*n* total: 50/812	—	12/271	14/271	24/270	—
Model 1	1.02 (0.94, 1.11)	1 (ref)	1.25 (0.55, 2.82)	2.04 (0.96, 4.34)	0.055
Model 2	1.03 (0.94, 1.13)	1 (ref)	1.13 (0.48, 2.65)	2.14 (0.97, 4.71)	0.047

Odds ratio (OR) represents changes in preeclampsia risk per 50 g change in ultraprocessed food (UPF) consumption and each tertile of change, compared with tertile 1 (lowest consumption), the reference category. Model 1 was controlled for age (<40; ≥40); socioeconomic status (low vs. high); baseline energy intake (kcal/d); ethnicity (White vs. no White); prepregnancy BMI (<30/≥30 km/m^2^); and intervention arm. Model 2 was further adjusted by nulliparity (yes/no); smoking during pregnancy (yes/no); and previous hypertensive disorder (yes/no) and the use of assisted reproductive technologies (yes/no). To assess the linear trend (*P*-trend) across tertiles of UPF, the mean value was assigned to each tertile.

Regarding the association of UPF with overall preeclampsia, in the stratified analysis by maternal prepregnancy BMI and socioeconomic status, no interaction was observed (Supplementary Table 4). No statistically significant associations were found between UPF consumption and other perinatal outcomes (Supplementary Table 5).

**FIGURE 2:**
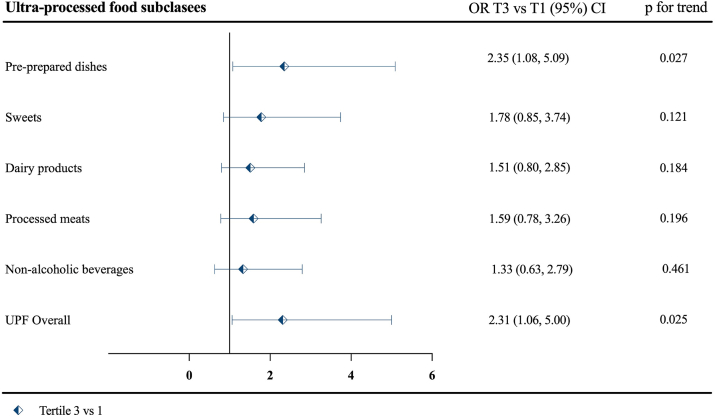
Association between changes in ultraprocessed food (UPF) subclass consumption during pregnancy and the occurrence of preeclampsia. Odds ratio (OR) represents changes in preeclampsia risk in tertile 3 of change in UPF consumption (high consumption), compared with tertile 1, the reference category. Analyses were performed using logistic regression models adjusted by age (<40; ≥40); socioeconomic status (low vs. high); baseline energy intake (kcal/d); ethnicity (White vs. no White); prepregnancy BMI (<30/≥30 kg/m^2^); intervention arm; nulliparity (yes/no); smoking during pregnancy (yes/no); previous hypertensive disorder (yes/no); and the use of assisted reproductive technologies (yes/no). To assess the linear trend (*P*-trend) across tertiles of UPF, the mean value was assigned to each tertile.

Regarding UPF subclasses, the risk of preeclampsia was associated with the consumption of pre-prepared dishes [OR_T3 compared with T1_ 2.35 (95% CI: 1.08, 5.09), *P*-trend = 0.027] but not with sweets [OR_T3 compared with T1_ 1.78 (0.85, 3.74), *P*-trend = 0.121] (Figure 2 and Supplementary Table 6).

### Maternal UPF consumption and diet quality

According to maternal key food intake, females with a higher UPF consumption showed increased intake of refined cereals, pastries, cakes, sweets, and processed meats. On the basis of the NOVA classification, participants with a higher consumption of UPF (increase intake during pregnancy) exhibited a significant reduction in the consumption of unprocessed or minimally processed (NOVA 1) intake [T3 compared with T1 mean difference −193.8 g/d (95% CI: −267.1, −120.5), *P* < 0.001], no significant changes were observed for NOVA 2 and NOVA 3 groups. Regarding maternal key food intake, females with higher UPF consumption had greater intakes of refined cereals, pastries, cakes, sweets, and processed meats (Supplementary Table 7). These females also exhibited higher overall caloric intake, along with greater consumption of SFAs, trans fatty acids, and carbohydrates during pregnancy (Supplementary Table 8).

Supporting these findings, an isotope mass ratio analysis conducted on a subsample of 531 participants revealed a significant association between isotopic nitrogen biomarkers (δ^15^ N ‰) and UPF consumption across tertiles, with corresponding δ^15^ N ‰ values. Specifically, positive trends were observed for the consumption of sweets and pastries, and dairy products. In addition, we also observed a tendency for higher N% values in relation to increased consumption of processed meats and nonalcoholic beverages. However, after adjusting by FDR, all trends disappeared (Table 3).

**TABLE 3:** Association between changes in total ultraprocessed food (UPF) consumption and subclasses during pregnancy and carbon and nitrogen isotopes ratios

	Changes in total UPF consumption and UPF subclasses (final vs. baseline)					*P* value^2^
	All females *n* = 531 (100)	Tertile 1 173 (32.6)	Tertile 2 170 (32.0)	Tertile 3 188 (35.6)	*P* value^1^	
Nitrogen %						
Ultraprocessed foods (g/d) (NOVA 4)	10.71 ± 1.08	10.67 ± 1.05	10.70 ± 0.79	10.77 ± 1.33	0.644	0.813
Dairy products (g/d)	10.71 ± 1.08	10.72 ± 1.10	10.84 ± 0.73	10.66 ± 1.10	0.620	0.813
Processed meats (g/d)	10.71 ± 1.08	10.61 ±1.11	10.67 ± 1.12	10.86 ± 0.98	0.076	0.348
Pre-prepared dishes (g/d)	10.71 ± 1.08	10.71 ± 0.75	10.71 ± 0.78	10.66 ± 1.52	0.696	0.835
Sweets (g/d)	10.71 ± 1.08	10.67 ± 1.10	10.73 ± 0.81	10.74 ± 1.26	0.797	0.911
Nonalcoholic beverage (g/d)	10.71 ± 1.08	10.75 ± 0.73	10.57 ± 1.14	10.85 ± 1.45	0.067	0.348
δ15/14N AIR						
Ultraprocessed foods (g/d) (NOVA 4)	7.89 ± 0.72	7.78 ± 0.89	7.96 ± 0.66	7.94 ± 0.55	0.031	0.348
Dairy products (g/d)	7.89 ± 0.72	7.89 ± 0.82	8.10 ± 0.46	7.84 ± 0.56	0.087	0.348
Processed meats (g/d)	7.89 ± 0.72	7.88 ± 0.74	7.89 ± 0.84	7.90 ± 0.59	0.958	0.958
Pre-prepared dishes (g/d)	7.89 ± 0.72	7.89 ± 0.83	7.94 ± 0.62	7.84 ± 0.70	0.452	0.723
Sweets (g/d)	7.89 ± 0.72	7.78 ± 0.96	7.94 ± 0.64	7.95 ± 0.51	0.057	0.348
Nonalcoholic beverage (g/d)	7.89 ± 0.72	7.84 ± 0.77	7.92 ± 0.74	7.94 ± 0.59	0.355	0.723
Carbon % (SD)						
Ultraprocessed foods (g/d) (NOVA 4)	41.56 ± 3.89	41.39 ± 3.73	41.44 ± 2.74	41.88 ± 4.91	0.431	0.723
Dairy products (g/d)	41.56 ± 3.90	41.54 ± 4.16	42.36 ± 2.52	41.39 ± 3.63	0.327	0.723
Processed meats (g/d)	41.57 ± 3.90	41.17 ± 4.08	41.31 ± 3.72	42.20 ± 3.77	0.024	0.348
Pre-prepared dishes (g/d)	41.57 ± 3.90	41.67 ± 2.28	41.50 ± 2.59	41.52 ± 5.78	0.906	0.945
Sweets (g/d)	41.57 ± 3.90	41.36 ± 3.83	41.49 ± 2.89	41.82 ± 4.66	0.511	0.766
Nonalcoholic beverage (g/d)	41.57 ± 3.90	41.73 ± 2.28	41.08 ± 4.28	41.97 ± 5.36	0.103	0.353
δ13/12C V-PDB						
Ultraprocessed foods (g/d) (NOVA 4)	−22.63 ± 1.28	−22.50 ± 1.62	−22.68 ± 0.87	−22.74 ± 1.21	0.181	0.543
Dairy products (g/d)	−22.63 ± 1.29	−22.57 ± 1.39	−22.65 ± 0.85	−22.74 ± 1.16	0.383	0.723
Processed meats (g/d)	−22.63 ± 1.29	−22.55 ± 1.64	−22.69 ± 1.16	−22.67 ± 0.92	0.555	0.783
Pre-prepared dishes (g/d)	−22.63 ± 1.29	−22.55 ± 0.64	−22.76 ± 1.20	−22.59 ± 1.76	0.263	0.701
Sweets (g/d)	−22.63 ± 1.29	−22.55 ± 1.70	−22.74 ± 1.11	−22.61 ± 0.94	0.408	0.723
Nonalcoholic beverage (g/d)	−22.63 ± 1.29	−22.64 ± 1.00	−22.60 ± 1.73	−22.67 ± 0.94	0.894	0.945

*n* = 531. Values are presented as the means (SD). Changes in UPF consumption and subclasses were divided into tertiles of change from baseline to final visit, being tertile 1 the lowest consumption of UPF.

1 One-way analysis of variance was used to compare continuous variables across tertiles of UPF change.

2 False discovery rate–adjusted *P* value.

### Impact of maternal lifestyle intervention on UPF intake

At enrollment, total UPF consumption was similar in the 3 study treatment arms (*P* = 0.602). However, at the end of pregnancy, the Mediterranean diet intervention group exhibited a significant reduction in UPF intake compared with stress reduction and usual care groups [Mediterranean diet compared with usual care: mean difference −70.1 g/d (95% CI: −91.4, −48.8), *P* < 0.001; Mediterranean diet compared with stress reduction: mean difference −74.3 g/d (95% CI: −96.1, −52.5), *P* < 0.001] (Table 4).

**TABLE 4:** Changes in maternal ultraprocessed food consumption at baseline and final assessment according to intervention group

		Within-group mean changes				*P* value^4^	Between-group changes		
		Mediterranean diet	Stress reduction	Usual care	*P* value^3^		Mediterranean diet vs. usual care	Stress reduction vs. usual care	Mediterranean diet vs. stress reduction
		*n* = 292	*n* = 255	*n* = 273			Difference (95% CI)	Difference (95% CI)	Difference (95% CI)
Unprocessed and minimally processed foods (NOVA 1) (g/d)	Baseline^1^	2448 (503.1)	2480 (513.4)	2423 (494.5)			—	—	—
	Final^2^	2833 (24.5)	2547.9 (26.4)	2526 (25.3)	<0.001	<0.001	307.1 (237.9, 376.3)	21.6 (−50.3, 93.4)	285.5 (214.8, 356.2)
Processed culinary ingredients (NOVA 2) (g/d)	Baseline	80.7 (25.2)	82.4 (24.7)	83.6 (27.1)			—	—	—
	Final	83.3 (1.33)	83.7 (1.43)	83.0 (1.37)	0.940	0.94	0.32 (−3.43, 4.08)	0.70 (−3.20, 4.59)	−0.37 (−4.21, 3.46)
Processed food (NOVA 3) (g/d)	Baseline	201.3 (126.9)	208.6 (128.1)	210.3 (152.1)			—	—	—
	Final	224.1 (5.93)	204.8 (6.39)	198.3 (6.12)	0.007	0.01	25.9 (9.14, 42.6)	6.52 (−10.8, 23.9)	19.3 (2.25, 36.4)
Ultraprocessed foods (NOVA 4) (% energy)	Baseline^1^	20.5 (8.71)	20.1 (8.31)	20.8 (9.35)			—	—	—
	Final^2^	12.7 (0.39)	19.0 (0.42)	19.5 (0.40)	<0.001	<0.001	−6.73 (−7.83, −5.64)	−0.46 (−1.59, 0.68)	−6.28 (−7.39, −5.16)
Ultraprocessed foods (NOVA 4) (g/d)	Baseline	227.8 (150.3)	218.2 (119.7)	231.1 (175.9)			—	—	—
	Final	151.5 (7.57)	225.7 (8.15)	221.6 (7.81)	<0.001	<0.001	−70.1 (−91.4, −48.8)	4.14 (−18.0, 26.3)	−74.3 (−96.1, −52.5)
Dairy products (g/d)	Baseline	22.7 (36.8)	23.3 (45.7)	23.3 (35.0)			—	—	—
	Final	22.9 (2.89)	23.2 (3.09)	26.8 (2.98)	0.593	0.659	−3.89 (−12.0, 4.27)	−3.55 (−12.0, 4.87)	−0.32 (−8.63, 7.98)
Processed meats (g/d)	Baseline	29.6 (20.6)	31.9 (29.2)	27.6 (22.7)			—	—	—
	Final	29.2 (1.02)	27.7 (1.09)	27.2 (1.06)	0.388	0.485	1.94 (−0.94, 4.82)	0.51 (−2.47, 3.49)	1.43 (−1.49, 4.36)
Pre-prepared dishes (g/d)	Baseline	35.0 (25.9)	31.4 (19.9)	31.1 (21.1)			—	—	—
	Final	23.7 (0.99)	28.8 (1.06)	31.1 (1.03)	<0.001	<0.001	−7.36 (−10.1, −4.57)	−2.28 (−5.17, 0.61)	−5.08 (−7.92, −2.24)
Sweets (g/d)	Baseline	42.9 (33.6)	42.2 (30.3)	45.6 (33.9)			—	—	—
	Final	30.9 (1.48)	40.6 (1.58)	40.9 (1.52)	<0.001	<0.001	−10.1 (−14.2, −5.90)	−0.32 (−4.62, 3.98)	−9.74 (−14.0, −5.50)
Nonalcoholic beverage (g/d)	Baseline	69.7 (115.9)	65.4 (83.7)	75.5 (151.5)			—	—	—
	Final	32.2 (6.06)	74.9 (6.65)	67.7 (6.25)	<0.001	<0.001	−35.5 (−52.3, −18.4)	7.19 (−10.5, 24.8)	−42.7 (−60.1, −25.3)

1 Baseline values are observed means (SD).

2 Final values are baseline-adjusted (least-squares) means (SE).

3 Comparison among groups done with analysis of covariance analysis.

4 False discovery rate–adjusted *P* value.

## Discussion

In this secondary analysis of the IMPACT BCN trial, higher change in UPF consumption from the second to third trimester was associated with an increased risk of preeclampsia. Likewise, following a Mediterranean diet intervention during pregnancy was significantly associated with a reduction in the consumption of UPF, and the reduction in UPF consumption during pregnancy increased the overall quality of the diet. To our knowledge, this study is the first to prospectively assess the relationship between maternal UPF consumption and preeclampsia development using NOVA classification.

### Maternal UPF consumption and preeclampsia

Our findings suggest a significant association between higher maternal UPF consumption and an increased risk of preeclampsia, independent of confounders. These results suggest that UPF consumption may act as an independent risk factor for preeclampsia.

In addition, we observed a stronger association than previous reports [20], likely due to our high-risk study population and the ability to assess changes of UPF consumption between 2 pregnancy timepoints rather than relying on a single dietary measure [20].

Consistent with our findings, a recent meta-analysis reported a 22% increased risk of preeclampsia among individuals with higher UPF consumption [10]. Similarly, a prospective study in Denmark reported that a seafood-based diet high in vegetables was associated with lower risk of preeclampsia [OR: 0.79 (95% CI: 0.65, 0.97)]; meanwhile, Western diet, rich in meat, margarine, and white bread consumption, was associated with a higher risk [OR: 1.40 (95% CI: 1.11, 1.76)] [36]. These findings suggest that overall dietary quality may be an important modifiable factor affecting maternal cardiometabolic health. However, this highlights a key limitation of using the NOVA classification, because foods categorized as UPF often differ not only in processing methods or industrial ingredients but also in nutritional composition, making difficult to separate the independent contribution of food processing from that of nutritional attributes. In this context, our study highlights the importance of lifestyle interventions, particularly those that promote a healthy diet during pregnancy. Previous studies have reported that late-onset forms are related to pre-existing maternal cardiovascular risk factors, such as subclinical drivers of cardiovascular diseases, including endothelial dysfunction or elevated arterial stiffness [37]. Consistent with this evidence, we observed that higher UPF consumption was associated with an increased risk of preeclampsia, independent of maternal socioeconomic status or BMI. However, our CIs may reflect the limited number of preeclampsia cases in our follow-up and high-risk nature of the cohort.

Furthermore, our findings contrast from those in the United States and Canada, where almost 80% of total energy intake is from UPF consumption [38,39], with cookies, pastries, and sugar-sweetened beverages being the most consumed UPF. A recent cross-sectional study observed that pregnant females with higher UPF consumption had a lower intake of key nutrients during pregnancy, such as vitamin C, B6, and potassium, resulting in overall poorer diet quality [40], as observed in our study. They also observed that adherence to a traditional Mediterranean diet was associated with lower UPF consumption [40]. Given the high consumption of UPF, including sweets, in many populations, as well as their association with poorer diet quality, it is essential to explore how food processing modifies dietary biomarkers. Although previous research has primarily linked carbon isotope signatures (δ^13^C) to sweet consumption—largely because of the influence of sugars from C_4_ plants [41]—our results were inconclusive, probably because carbon isotopes is less sensitive in populations where cane sugar predominates over corn-based sweeteners. Furthermore, the isotope analyses were performed on a subsample, which limits the ability to detect dietary differences. Further research is needed to determine whether this pattern is consistent across different populations and dietary practices.

### Maternal UPF consumption and fetal outcomes

Our study found no significant associations between maternal UPF consumption and fetal outcomes, consistent with existing research. One study reported an association with insufficient birth weight, which disappeared after adjustment [42]. In childhood, every 10% increase in UPF consumption was associated with BMI z-scores and skinfold thickness measurements in boys, whereas no significant effects found in girls [43]. Further research is needed to fully elucidate the relationship of a nutrient-rich diet during pregnancy to support fetal growth and long-term child health.

### Underlying molecular mechanisms: preeclampsia pathogenesis and relationship with UPF consumption

Preeclampsia involves various pathways, including inflammation, systemic endothelial dysfunction [44], increased oxidative stress biomarkers, insulin resistance, dyslipidemia, and a disrupted gut microbiome [45]. Although the exact cause remains unclear, recent evidence indicates that dietary factors play a considerable role in its development. UPF consumption during pregnancy has been shown to increase oxidative stress markers [46,47]. Additionally, endothelial dysfunction, a key feature of preeclampsia [48], has been associated with dietary quality which could modify endothelial vasoconstriction. Sodium intake is a well-established risk factor for hypertension [49], and cardiovascular-related deaths. However, during pregnancy, the association between sodium intake and preeclampsia remains unclear [50]. Dietary factors may be more relevant in late-onset forms, which represent the majority of cases in our study, where the etiologic role of early placental implantation is likely to be less important than in early-onset forms, and they are thought to be mainly caused by an inadequate cardiovascular maternal adaptation to the hemodynamic demands of pregnancy [51].

### Strategies to reduce UPF consumption

An intervention based on the Mediterranean diet, as suggested in this study, may represent an easy-to-follow dietary approach to improve both obstetric and fetal outcomes [[23], [24], [25], [26], [27], [28], [29], [30], [31], [32], [33], [34], [35], [36], [37], [38], [39], [40], [41], [42], [43], [44], [45], [46], [47], [48], [49], [50], [51], [52]]. Specifically, we observed that an intervention involving a pregnancy-adapted Mediterranean diet reduced the intake of UPF, including both ultraprocessed and processed items, whereas significantly increasing the consumption of unprocessed or minimally processed foods. By definition, the Mediterranean diet emphasizes plant-based and unprocessed foods [53], and promoted a reduction in UPFs, particularly refined cereals, processed meats, carbonated and/or sugar-sweetened beverages, and pastries such as cookies, custards, sweets, and cakes [[22], [23], [24], [25], [26], [27], [28], [29], [30], [31], [32], [33], [34], [35], [36], [37], [38], [39], [40], [41], [42], [43], [44], [45], [46], [47], [48], [49], [50], [51], [52], [53], [54]].

### Strengths and limitations

Among strengths, this study is based on a randomized controlled trial framework, which represents a well-characterized population with a large sample size. Dietary information was collected by trained dietitians using standardized, validated dietary questionnaires for pregnancy, and for this study population, which showed good reproducibility and validity [24]. Nevertheless, we acknowledge some limitations. First, this is a secondary analysis of a randomized controlled trial. Post hoc analyses are considered valid but are subject to bias [55]. Second, prepregnancy diet and weight could not be assessed. Third, the use of the FFQ and NOVA classification may have led to a misclassification within NOVA groups because the FFQ used was not specifically designed to assess UPF consumption; however, it included a large variety of the most consumed foods in Spain (151 items) and UPF consumption was well represented by 38 different items. The classification was conducted by consensus among nutrition experts. Forth, the study included high-risk women and was conducted at a high-income setting, which limits the generalizability of the findings to other populations or settings. Fifth, the population was mainly comprised late-preeclampsia forms. Therefore, the conclusions of the study might be different for early-onset forms.

In conclusion, in this study of high-risk pregnancies, the change in maternal UPF consumption during pregnancy was associated with preeclampsia risk, which was predominately late-onset preeclampsia. Likewise, following a Mediterranean diet intervention during pregnancy significantly reduced the consumption of UPF, and a reduction in UPF consumption during pregnancy increased the overall quality of the diet. Future research should prioritize trials that assess UPF consumption as a primary exposure to validate these findings and to inform the development of targeted interventions and public health strategies that promote healthier dietary choices among pregnant females, to ensure the reduction of UPF consumption and lower preeclampsia risk.

## Author contributions

The authors’ responsibilities were as follows – EG, F Crispi, F Crovetto, EV, SC-B, RC, RE: design research; SC-B, F Crispi, F Crovetto, RE; AMR-L, ML, AN; LY, LB, EV, EG, RC: conducted research; ATD, LB, SC-B, F Crispi, F Crovetto, RC, RE: analyzed data; F Crovetto, RE: responsibility of the final content; ATD, LB, SC-B, F Crovetto, RE: writing—original draft preparation; and all authors: read and approved the final manuscript.

## Data availability

Data described in the manuscript, code book, and analytic code will be made available from the corresponding author on reasonable request.

## Funding

The project was partially funded by grants from Instituto de Salud Carlos III (ISCIII) (PI22/00684; PI22/00109; PI20/00246; PI22/00689; PI24/00127) cofunded by the European Union, AGAUR Departament de Recerca i Universitats de la Generalitat de Catalunya al Grup de Recerca de Medicina Maternofetal i Reproductiva (Codi: 2021-SGR-01422), “la Caixa” Foundation (LCF/PR/ GN18/10310003); the Cerebra Foundation for the Brain Injured Child (Carmarthen, Wales, United Kingdom) and the Fundación Mutua Madrileña (AP16002/2024; AP180722022). ATD was directly supported by a personal research grant from the ISCIII (FI23/00316). LB was supported by a research grant from the Instituto de Salud Carlos III (CM21/00058). MG was supported by a research grant post-FSE Starting from the Hospital Sant Joan de Déu. AN has received support from a fellowship from “la Caixa” Foundation, Doctoral INPhINIT Retaining fellowship (LCF/BQ/DR19/11740018). LY was supported by the fellowship program “Biomedicine International training research programme for excellent clinician-scientist” (BITRECS) by Barcelona Clinic Hospital and funded by the “la Caixa” Foundation (code num. LCF/PR/SP23/52950012). IC has received a personal grant from ISCIII (CM23/00118), cofunded by the European Union. F Crispi has received support from Hospital Clinic (programa Intensificacions) and Fundació Occident (Premi a la Investigació Jesus Serra) (Spain). SC-B has received support from Instituto de Salud Carlos III, through the competitive Sara Borrell fellowship (code CD24/00244) cofunded by European Union. RE has received support from the Instituto de Salud Carlos III (AC19/00100), as part of the FoodPhyt project, under the umbrella of the European Joint Programming Initiative “A Healthy Diet for a Healthy Life” (JPI HDHL) (2019–02201). JO’S was supported by the NSW Health Early-Mid Career Fellowship and Clinician-Scientist Awards (DOH1003; DOH1006); National Heart Foundation Future Leader Fellowship (NHF104853) and NHMRC-MRFF Cardiovascular Health Mission (107180). INSA-UB is María de Maeztu Unit of Excellence (grant CEX2021-001234-M funded by MICIN/AEI/FEDER, UE). The funders had no role in the design and conduct of the study; collection, management, analysis, and interpretation of the data; preparation, review, or approval of the manuscript; and decision to submit the manuscript for publication.

## Conflict of interest

RE reports grants from the Fundación Dieta Mediterránea (Spain), and Cerveza y Salud (Spain), and personal fees for given lectures from Brewers of Europe (Belgium), the Fundación Cerveza y Salud (Spain), Pernaud-Ricard (Mexico), Instituto Cervantes (Alburquerque, United States), Instituto Cervantes (Milan, Italy), Instituto Cervantes (Tokyo, Japan), Lilly Laboratories (Spain), and the Wine and Culinary International Forum (Spain), as well as nonfinancial support for the organization of a National Congress on Nutrition and feeding trials with products from Grand Fountain and Uriach Laboratories (Spain). EV has received grants and served as consultant, advisor or CME speaker for the following entities: AB-Biotics, Abbott, AbbVie, Adamed, Alcediag, Angelini, Biogen, Beckley-Psytech, Biohaven, Boehringer-Ingelheim, Casen-Recordati, Celon Pharma, Compass, Dainippon Sumitomo Pharma, Esteve, Ethypharm, Ferrer, Gedeon Richter, GH Research, Glaxo-Smith Kline, HMNC, Idorsia, Johnson & Johnson, Lundbeck, Luye Pharma, Medincell, Merck, Mitsubishi Tanabe Pharma, Newron, Novartis, Organon, Orion Corporation, Otsuka, Roche, Rovi, Sage, Sanofi-Aventis, Sunovion, Takeda, Teva, and Viatris, outside the submitted work. The other authors report no conflicts of interest.
