# Supplementary material for: Ultraprocessed foods consumption and risk of preeclampsia: a secondary analysis of the improving mothers for a better prenatal care trial Barcelona (IMPACT BCN) randomized clinical trial
Source: Am J Clin Nutr. 2026 Mar 2;123(5):101255. doi: 10.1016/j.ajcnut.2026.101255 (PMC13197901; doi:10.1016/j.ajcnut.2026.101255)
Supplement: Multimedia component 1 [file mmc1.docx]

**Ultra-processed foods consumption and risk of preeclampsia. A secondary analysis of the IMPACT BCN randomized clinical trial. TREJO-DOMÍNGUEZ Alejandra**

***Supplementary material***

**Supplementary Table S1.** Distribution of items of food frequency questionnaire into four groups according to the degree of their processing established by NOVA classification system.

| **NOVA 1:** Unprocessed or minimally processed foods | Rice (whole-grain and refined), pasta, noodles, macaroni, couscous (whole-grain and refined), quinoa, chia, legumes (lentils, beans, chickpeas, peas), fresh vegetables (chard, spinach, cabbage, cauliflower , broccoli, lettuce, raw tomato, carrot, pumpkin, green beans, eggplant, zucchini, peppers, asparagus, onion, garlic, mushrooms and boiled potatoes) *gazpacho*, fresh fruit (orange, banana, apple, strawberries, cherries, kiwi, pineapple, grapes, dates), nuts (almonds, hazelnuts, walnuts, pistachios), milk (whole-fat, semi-skimmed and skimmed), yogurt (whole-fat and skimmed), eggs, fresh fish and seafoods (oysters, clams, mussels, squid, octopus, crustaceans), meats (chicken, turkey, beef, pork, lamb, rabbit), avocado and herbs (parsley, thyme, oregano and pepper), water, infusions, tea, coffee and natural fruit juice. |
| --- | --- |
| **NOVA 2:** Processed culinary ingredients | Vegetable oils (refined and extra-virgin olive oil, oils from sunflower seeds, corn, soybean and coconut), butter, lard, salt, honey and sugar. |
| **NOVA 3:** Processed foods | Whole-grain bread, home-made French fries, fruits in syrup, chocolate (>70% cocoa), condensed milk, cream or milk cream, cottage cheese or curd, cheeses (cured, semi-cured, cottage and fresh), canned fish, salt-curing and drying fish, cured ham, olives, decaffeinated coffee. |
| **NOVA 4**: Ultra-processed foods and drink products | White bread, breakfast cereals, cookies (home-made and industrial), commercial pastries (croissant, *ensaimada*, donuts, muffins, cakes, *churros*), ice cream, petit suisse, creamy cheese spreads, margarine, custard, flan, pudding, milkshakes, surimi, ham, bacon, processed meat (*chorizo*, sausage, blood sausage, *mortadella, sobrasada*, pâtés, hamburger or meatballs, ham), milk chocolate, sugary cocoa powder, confectionery (marzipan and nougat), pre-prepared dishes (croquettes, *empanadillas*, pizza), instant soups, mustard, mayonnaise, packed fried tomato sauce or ketchup, potato chips, savory packed snacks, sweeteners, soft drinks (sugar- and artificially-sweetened), commercial fruit juices, non-alcoholic beer. |

**Supplementary Table S2.** Distribution of items into five ultra-processed food (UPF) subgroups according to food characteristics established by NOVA classification system.

| **Group 1**: Dairy products | Ice cream and flan, milkshakes, cheese spreads, custard, pudding. |
| --- | --- |
| **Group 2**: Processed meats | Surimi, hamburgers, meat balls, ham, bacon, *chorizo*, *mortadella*, sausages, blood sausage, *sobrasada*, pâtés and foie-gras. |
| **Group 3**: Pre-prepared dishes | Croquettes, *empanadillas*, pizza, instant soups and creams, mustard, margarine, mayonnaise, ketchup, packed fried tomato sauce, potato chips, savory packed snacks, and popcorn. |
| **Group 4**: Sweets | Breakfast cereals, white bread, cookies (home-made and industrial), commercial pastries (croissant, *ensaimada*, donuts, muffins, cakes, *churros*), confectionery (marzipan and nougat), milk chocolate, sugary cocoa powder, and artificial sweeteners. |
| **Group 5**: Non-alcoholic beverage | Soft drinks (sugar- and artificially sweetened), energy drinks or isotonic drinks, commercial fruit juices and non-alcoholic beer. |

**Supplementary Table S3.** Characteristics of the study population according to baseline consumption of ultra-processed foods (UPF).

|  |  | **UPF consumption (baseline)** | | |
| --- | --- | --- | --- | --- |
|  | **All females** | **UPF Tertile 1**  105.1 (33.1) g/d | **UPF Tertile 2**  190.6 (25.4) g/d | **UPF Tertile 3**  383.2 (161.7) g/d |
| **N (%)** | **812 (100)** | **273 (33.6)** | **269 (33.2)** | **270 (33.2)** |
| **MATERNAL BASELINE CHARACTERISTICS** | | | | |
| Age (years) | 37.3 (4.6) | 37.9 (4.8) | 37.1 (4.2) | 36.7 (4.7) |
| Ethnicity  - White  - Latin  - Asian  - Afro-American  - Others | 673 (82.9)  103 (12.7)  12 (1.5)  10 (1.2)  14 (1.7) | 234 (85.7)  29 (10.6)  0 (0.0)  3 (1.1)  7 (2.5) | 226 (84.0)  30 (11.1)  9 (3.3)  0 (0.0)  4 (1.4) | 213 (78.8)  44 (16.3)  3 (1.1)  7 (2.6)  3 (1.1) |
| Smoking habit  - No  - Stop during pregnancy  - Yes | 658 (81.0)  102 (12.5)  52 (6.4) | 231 (84.6)  34 (12.4)  8 (2.9) | 227 (84.3)  27 (10.0)  15 (5.5) | 200 (74.0)  41 (15.1)  29 (10.7) |
| Educational level  - Primary school  - Secondary school  - University | 33 (7.8)  221(52.7)  165 (39.3) | 14 (5.1)  56 (20.5)  203 (74.3) | 9 (3.3)  70 (26.0)  190 (70.6) | 10 (3.7)  95 (35.1)  165 (61.1) |
| Employment status  - Student  - Employed  - Autonomous  - Housekeeper  - Unemployed | 7 (0.8)  669 (82.3)  60 (7.3)  23 (2.8)  53 (6.5) | 3 (1.1)  223 (81.6)  26 (9.5)  5 (1.8)  16 (5.8) | 2 (7.4)  228 (84.7)  18 (6.6)  7 (2.6)  2 (0.7) | 2 (0.74)  218 (80.7)  16 (5.9)  11 (4.0)  23 (8.5) |
| Nulliparity | 477 (58.7) | 169 (61.9) | 154 (57.2) | 154 (57.0) |
| Use of assisted reproductive technologies | 217 (26.7) | 83 (30.4) | 67 (24.9) | 67 (24.8) |
| Previous hypertensive disorder | 31 (3.8) | 8 (2.9) | 13 (4.8) | 10 (3.7) |
| **MATERNAL MEASUREMENTS DURING PREGNANCY** | | | | |
| Weight (Kg)  - Preconceptional  - 19-23 weeks’ gestation | 63.5 (12.8)  69.5 (12.2) | 62.3 (12.6)  68.4 (12.0) | 64.3 (13.0)  70.0 (12.2) | 63.9 (12.7)  70.0 (12.3) |
| Body Mass Index (kg/m^2^)  - Preconceptional  - 19-23 weeks’ gestation | 23.7 (4.6)  26.0 (4.5) | 23.2 (4.5)  25.5 (4.3) | 24.2 (4.8)  26.3 (4.8) | 23.9 (4.4)  26.2 (4.3) |
| Blood pressure (mmHg) at 19-23 weeks’ gestation  - Systolic  - Diastolic | 105.2 (11.8)  67.6 (8.5) | 105.1(12.2)  67.3 (8.3) | 105.9 (12.9)  67.9 (8.8) | 104.7 (10.8)  67.6 (8.3) |

Values are presented as the means (SD) for continuous variables and n (%) for categorical variables. Changes in UPF consumption was divided into tertiles of change in UPF consumption from baseline to final visit, being tertile 1 the lowest consumption of UPF.

**Supplementary Table S4.** Association of changes in ultra-processed food (UPF) consumption during pregnancy and the occurrence of preeclampsia stratified by maternal pre-pregnancy body mass index and socioeconomic status.

| **Risk factor** | **Cases PE/N** | **Multivariate OR (95% CI)**  **T3 *vs.* T1** | **P for trend** | **P for interaction** |
| --- | --- | --- | --- | --- |
| **Pre-pregnancy BMI** |  |  |  | 0.377 |
| Underweight | 1/28 | NA | NA |  |
| Normal weight | 29/530 | 2.95 (0.90, 9.65) | 0.067 |  |
| Overweight | 15/164 | 1.31 (0.31, 5.58) | 0.617 |  |
| Obesity | 9/88 | 2.68 (0.23, 31.6) | 0.468 |  |
| **Socioeconomic status** |  |  |  | 0.388 |
| Low | 4/33 | NA | NA |  |
| Medium | 21/250 | 0.56 (0.12, 2.64) | 0.185 |  |
| High | 29/527 | 2.33 (0.79, 6.90) | 0.123 |  |

Odds ratio (OR) represents changes in preeclampsia risk in tertile 3 of change in ultra-processed food (UPF) consumption, compared to tertile1, the reference category. Models were adjusted for age (<40; ≥40); baseline energy intake (kcal/day); ethnicity (White vs. no White); intervention arm; nulliparity (yes/no); smoking during pregnancy (yes/no); and previous hypertensive disorder (yes/no), the use of assisted reproductive technologies (yes/no), socioeconomic status (low vs. high), only in the stratified analysis for pre-pregnancy body mass index (BMI), and pre-pregnancy body mass index (<30/≥30 kg/m^2^), only in the stratified analysis for socioeconomic status;. To assess the linear trend (p for trend) across tertiles of UPF, the mean value was assigned to each tertile.

**Supplementary Table S5.** Association of changes in total UPF consumption during pregnancy and perinatal outcomes.

| **Perinatal Outcomes** | **OR (95% CI)** | | | Adjusted p for trend |
| --- | --- | --- | --- | --- |
|  | **UPF Tertile 1**  (-1342, -57.2g/d) | **UPF Tertile 2**  (-57.2, 10.5 g/d) | **UPF Tertile 3**  (10.5, 1453 g/d) |  |
| **Small for Gestational Age newborn** |  |  |  |  |
| N cases/N total: 125 / 812 (15.3%) | **45/271** | **41/271** | **39/270** |  |
| Model 1 | 1 (ref) | 0.83 (0.51, 1.33) | 0.78 (0.48, 1.27) | 0.313 |
| Model 2 | 1 (ref) | 0.82 (0.51, 1.33) | 0.79 (0.48, 1.29) | 0.344 |
| **Severe Small for Gestational Age newborn** |  |  |  |  |
| N cases/N total: 51 / 812 (6.8%) | **17/271** | **19/271** | **15/270** |  |
| Model 1 | 1 (ref) | 0.97 (0.48, 1.94) | 0.77 (0.36, 1.62) | 0.488 |
| Model 2 | 1 (ref) | 0.95 (0.46, 1.96) | 0.82 (0.38, 1.75) | 0.605 |
| **Preterm birth** |  |  |  |  |
| N cases/N total: 37 / 812 (4.5%) | **9/271** | **11/271** | **17/270** |  |
| Model 1 | 1 (ref) | 1.39 (0.55, 3.54) | 2.04 (0.85, 4.89) | 0.103 |
| Model 2 | 1 (ref) | 1.30 (0.50, 3.34) | 1.96 (0.81, 4.73) | 0.124 |
| **Adverse Perinatal Outcome** |  |  |  |  |
| N cases/N total: 164 / 812 (20.1%) | **53/271** | **51/271** | **60/270** |  |
| Model 1 | 1 (ref) | 0.97 (0.62, 1.51) | 1.10 (0.71, 1.71) | 0.643 |
| Model 2 | 1 (ref) | 0.94 (0.60, 1.48) | 1.10 (0.71,1.72) | 0.648 |

Odds ratio (OR) represents changes in perinatal outcomes risk in each tertile of change in ultra-processed food (UPF) consumption, compared to tertile1, the reference category. **Model 1** was adjusted by age (<40; ≥40); socioeconomic status (low vs. high); baseline energy intake (kcal/day); ethnicity (White vs. no White); pre-pregnancy body mass index (<30/≥30 kg/m^2^); and intervention arm. **Model 2** was additionally adjusted by nulliparity (yes/no); smoking during pregnancy (yes/no); and previous hypertensive disorder (yes/no) and the use of assisted reproductive technologies (yes/no). To assess the linear trend (p for trend) across tertiles of UPF, the mean value was assigned to each tertile.

Outcomes definition: 1) Small for Gestational Age: Birth weight below the 10^th^ percentile for gestational age; 2) Severe small for gestational age (SGA): birth weight <3^rd^ centile; 3) Preterm birth, defined as delivery <37 weeks’ gestation, and 4) Adverse Perinatal Outcome, defined according to the presence of any of the following neonatal measures: preterm birth, preeclampsia, perinatal mortality, severe SGA, metabolic acidosis or, major neonatal morbidity

**Supplementary Table S6.** Association between changes in ultra-processed food (UPF) subclasses consumption during pregnancy and the occurrence of preeclampsia.

|  | **OR for overall preeclampsia (95% Confidence Interval)** | | | Adjusted p for trend |
| --- | --- | --- | --- | --- |
|  | **UPF Tertile 1** | **UPF Tertile 2** | **UPF Tertile T3** |  |
| **Pre prepared dishes** | **12/271** | **16/272** | **26/269** |  |
| Model 1 | 1 (ref) | 1.39 (0.64, 3.04) | 2.06 (1.00, 4.24) | 0.044 |
| Model 2 | 1 (ref) | 1.49 (0.65, 3.42) | 2.35 (1.08, 5.09) | 0.027 |
| **Sweets** | **14/272** | **16/271** | **24/269** |  |
| Model 1 | 1 (ref) | 1.19 (0.56, 2.54) | 1.78 (0.87, 3.62) | 0.101 |
| Model 2 | 1 (ref) | 1.27 (0.58, 2.81) | 1.78 (0.85, 3.74) | 0.121 |
| **Dairy products** | **26/489** | **6/67** | **22/256** |  |
| Model 1 | 1 (ref) | 1.76 (0.69, 4.49) | 1.48 (0.81, 2.72) | 0.181 |
| Model 2 | 1 (ref) | 2.03 (0.76, 5.44) | 1.51 (0.80, 2.85) | 0.184 |
| **Processed meats** | **18/271** | **15/271** | **21/270** |  |
| Model 1 | 1 (ref) | 0.82 (0.40, 1.69) | 1.29 (0.66, 2.52) | 0.439 |
| Model 2 | 1 (ref) | 0.85 (0.40, 1.84) | 1.59 (0.78, 3.26) | 0.196 |
| **Nonalcoholic beverages** | **18 /321** | **19/289** | **17/202** |  |
| Model 1 | 1 (ref) | 1.23 (0.62, 2.44) | 1.33 (0.65, 2.70) | 0.424 |
| Model 2 | 1 (ref) | 1.09 (0.53, 2.21) | 1.33 (0.63, 2.79) | 0.461 |

Odds ratio (OR) represents changes in preeclampsia risk in each tertile of change in each group of ultra-processed food (UPF) consumption, compared to tertile 1, the reference category. **Model 1** was adjusted by age (<40; ≥40); socioeconomic status (low vs. high); baseline energy intake (kcal/day); ethnicity (White vs. no White); pre-pregnancy body mass index (<30/≥30 kg/m^2^); and intervention arm. **Model 2** was additionally adjusted by nulliparity (yes/no); smoking during pregnancy (yes/no); and previous hypertensive disorder (yes/no) and the use of assisted reproductive technologies (yes/no). To assess the linear trend (p for trend) across tertiles of UPF, the mean value was assigned to each tertile.

**Supplementary Table S7.** Changes in maternal key food intake and Mediterranean diet adherence at baseline and final assessment according to tertiles of maternal ultra-processed food (UPF) consumption.

|  |  | **UPF Tertile 1**  (-1342, -57.2g/d) | **UPF Tertile 2**  (-57.2, 10.5 g/d) | **UPF Tertile 3**  (10.5, 1453 g/d) |  | **Tertile 3 *vs.* Tertile 1** |
| --- | --- | --- | --- | --- | --- | --- |
|  |  |  |  |  | *P*3 | *Difference (95% CI)* |
| Extra virgin olive oil – g/d | Baseline^1^ | 30.3 (19.6) | 36.6 (17.6) | 34.6 (19.6) |  |  |
|  | Final^2^ | 42.2 (0.96) | 39.7 (0.96) | 37.5 (0.96) | 0.002 | -4.71 (-7.37, -2.04) |
| Refined olive oil – g/d | Baseline | 9.50 (15.8) | 6.49 (13.5) | 6.81 (14.2) |  |  |
|  | Final | 4.62 (0.73) | 4.74 (0.73) | 6.39 (0.73) | 0.159 | 1.77 (-0.26, 3.79) |
| Total nuts – g/d | Baseline | 15.1 (15.6) | 19.6 (20.1) | 20.7 (20.0) |  |  |
|  | Final | 26.5 (1.09) | 24.2 (1.08) | 19.8 (1.09) | <0.001 | -6.69 (-9.73, -3.66) |
| Vegetables – g/d | Baseline | 268.9 (117.0) | 293.8 (117.5) | 284.3 (128.9) |  |  |
|  | Final | 316.5 (6.18) | 294.4 (6.13) | 284.7 (6.15) | <0.001 | -31.8 (-48.8, -14.7) |
| Legumes – g/d | Baseline | 47.4 (37.0) | 53.2 (38.2) | 51.9 (36.0) |  |  |
|  | Final | 67.4 (2.30) | 60.7 (2.31) | 54.9 (2.33) | <0.001 | -12.5 (-19.0, -6.13) |
| Fruits – g/d | Baseline | 309.7 (166.9) | 340.0 (166.0) | 312.8 (158.2) |  |  |
|  | Final | 359.8 (9.03) | 344.1 (9.07) | 333.1 (9.07) | 0.111 | -26.6 (-51.7, -1.58) |
| Refined cereals – g/d | Baseline | 78.9 (48.4) | 54.4 (38.8) | 55.4 (39.3) |  |  |
|  | Final | 30.2 (2.15) | 49.9 (2.14) | 64.3 (2.13) | <0.001 | 34.1 (28.0, 40.1.) |
| Whole grain cereals – g/d | Baseline | 30.5 (42.5) | 40.7 (34.3) | 43.0 (40.0) |  |  |
|  | Final | 56.5 (2.15) | 48.8 (2.13) | 37.6 (2.15) | <0.001 | -18.9 (-24.9, -13.0) |
| Fish or seafood – g/d | Baseline | 70.2 (40.2) | 71.8 (40.2) | 71.2 (40.7) |  |  |
|  | Final | 86.1 (2.35) | 81.1 (2.34) | 79.5 (2.37) | 0.120 | -6.57 (-13.1, -0.02) |
| Fat fish – g/d | Baseline | 13.1 (14.7) | 16.0 (16.0) | 14.6 (16.1) |  |  |
|  | Final | 23.1 (1.12) | 23.4 (1.12) | 20.8 (1.12) | 0.217 | -2.21 (-5.32, 0.89) |
| Lean meat – g/d | Baseline | 72.3 (35.8) | 68.3 (36.0) | 65.9 (36.5) |  |  |
|  | Final | 74.0 (1.96) | 70.7 (1.95) | 73.5 (1.98) | 0.430 | -0.55 (-6.01, 4.90) |
| Red meat – g/d | Baseline | 50.5 (32.2) | 42.5 (31.6) | 45.9 (32.3) |  |  |
|  | Final | 42.8 (1.56) | 41.3 (1.57) | 45.8 (1.57) | 0.117 | 3.04 (-1.30, 7.38) |
| Processed meat – g/d | Baseline | 39.8 (31.9) | 31.5 (22.1) | 28.7 (23.1) |  |  |
|  | Final | 28.5 (1.12) | 29.9 (1.12) | 33.7 (1.12) | 0.003 | 5.20 (2.07, 8.33) |
| Pastries, cakes, or sweets – g/d | Baseline | 49.1 (41.9) | 33.3 (26.9) | 32.6 (26.8) |  |  |
|  | Final | 23.8 (1.58) | 32.0 (1.57) | 43.8 (1.58) | <0.001 | 20.0 (15.6, 24.4) |
| Dairy products – g/d | Baseline | 345.6 (194.5) | 294.6 (205.1) | 336.3 (202.7) |  |  |
|  | Final | 431.3 (12.0) | 371.3 (12.1) | 376.7 (12.1) | <0.001 | -54.7 (-88.1, -21.3) |
| Processed food (NOVA 3) – g/d | Baseline | 189.9 (125.6) | 212.9 (137.4) | 217.0 (143.6) |  |  |
|  | Final | 200.7 (6.18) | 211.9 (6.16) | 216.0 (6.18) | 0.198 | 15.2 (-1.94, 32.4) |
| Processed culinary ingredients (NOVA 2) – g/d | Baseline | 82.4 (26.8) | 81.1 (24.1) | 83.0 (26.1) |  |  |
|  | Final | 84.2 (1.38) | 82.7 (1.38) | 83.0 (1.38) | 0.728 | -1.18 (-5.00, 2.64) |
| Unprocessed and minimally processed foods (NOVA 1) – g/d | Baseline | 2410 (542.1) | 2476 (475.6) | 2464 (489.2) |  |  |
|  | Final | 2745 (26.4) | 2632 (26.4) | 2551 (26.4) | <0.001 | -193.8 (-267.1, -120.5) |
| Mediterranean diet score | Baseline | 6.94 (2.41) | 8.22 (2.43) | 8.01 (2.55) |  |  |
|  | Final | 10.9 (0.17) | 9.57 (0.17) | 8.21 (0.17) | <0.001 | -2.69 (-3.17, -2.20) |

^1^Baseline values are observed means (SD). ^2^Final values are baseline-adjusted (least-squares) means (SE) and comparison among groups done with ANCOVA analysis. ^3^ANCOVA analysis

**Supplementary Table S8.** Changes in maternal nutrient intake at baseline and final assessment by tertiles of maternal ultra-processed food (UPF) consumption

|  |  | **UPF Tertile 1**  (-1342, -57.2g/d) | **UPF Tertile 2**  (-57.2, 10.5 g/d) | **UPF Tertile 3**  (10.5, 1453 g/d) |  | **Tertile 3 vs Tertile 1** |
| --- | --- | --- | --- | --- | --- | --- |
|  |  |  |  |  | *P*3 | *Difference (95% CI)* |
| Energy – kcal/d | Baseline^1^ | 2469 (487.4) | 2357 (415.5) | 2364 (432.8) |  |  |
|  | Final^2^ | 2391 (23.5) | 2439 (23.5) | 2526 (23.5) | <0.001 | 135.0 (69.7, 200.4) |
| Protein – kcal/d | Baseline | 105.0 (22.4) | 99.9 (23.6) | 99.8 (22.5) |  |  |
|  | Final | 108.5 (1.34) | 106.2 (1.34) | 107.1 (1.34) | 0.473 | -1.35 (-5.08, 2.37) |
| Carbohydrate – g/d | Baseline | 227.3 (58.7) | 207.0 (45.8) | 209.3 (53.3) |  |  |
|  | Final | 197.1 (2.44) | 209.4 (2.43) | 225.1 (2.43) | <0.001 | 27.9 (21.1, 34.7) |
| Fiber – g/d | Baseline | 31.1 (9.51) | 33.8 (10.4) | 33.1 (10.5) |  |  |
|  | Final | 35.1 (0.51) | 34.7 (0.51) | 32.8 (0.51) | 0.004 | -2.23 (-3.63, -0.82) |
| Total fat – g/d | Baseline | 126.5 (29.5) | 125.4 (27.2) | 125.2 (27.4) |  |  |
|  | Final | 129.4 (1.51) | 130.8 (1.51) | 132.9 (1.51) | 0.255 | 3.51 (-0.68, 7.70) |
| SFA – g/d | Baseline | 35.2 (9.60) | 33.3 (9.35) | 33.1 (8.38) |  |  |
|  | Final | 33.1 (0.48) | 34.7 (0.48) | 36.3 (0.48) | <0.001 | 3.20 (1.86, 4.54) |
| MUFA – g/d | Baseline | 60.5 (15.4) | 61.4 (13.9) | 60.7 (14.9) |  |  |
|  | Final | 62.6 (0.76) | 63.0 (0.76) | 63.8 (0.76) | 0.524 | 1.19 (-0.90, 3.29) |
| PUFA – g/d | Baseline | 21.4 (7.09) | 21.4 (6.80) | 22.2 (7.79) |  |  |
|  | Final | 24.0 (0.43) | 23.5 (0.43) | 23.0 (0.43) | 0.276 | -0.97 (-2.17, 0.22) |
| α-Linoleic acid – g/d | Baseline | 14.1 (5.44) | 13.8 (4.84) | 14.6 (5.97) |  |  |
|  | Final | 16.0 (0.32) | 15.5 (0.32) | 15.0 (0.32) | 0.085 | -1.01 (-1.90, -0.12) |
| α-Linolenic acid – g/d | Baseline | 1.34 (0.58) | 1.37 (0.57) | 1.38 (0.61) |  |  |
|  | Final | 1.82 (0.04) | 1.66 (0.04) | 1.53 (0.04) | <0.001 | -0.29 (-0.41, -0.16) |
| EPA – g/d | Baseline | 0.14 (0.10) | 0.16 (0.10) | 0.15 (0.10) |  |  |
|  | Final | 0.21 (0.01) | 0.20 (0.01) | 0.19 (0.01) | 0.237 | -0.02 (-0.03, 0.00) |
| DHA – g/d | Baseline | 0.29 (0.23) | 0.34 (0.25) | 0.32 (0.25) |  |  |
|  | Final | 0.45 (0.02) | 0.44 (0.02) | 0.40 (0.02) | 0.124 | -0.04 (-0.09, 0.00) |
| *Trans*-FA – g/d | Baseline | 1.80 (1.14) | 1.49 (1.05) | 1.55 (1.09) |  |  |
|  | Final | 1.19 (0.05) | 1.41 (0.05) | 1.65 (0.05) | <0.001 | 0.46 (0.31, 0.60) |
| Cholesterol – mg/d | Baseline | 340.3 (87.0) | 318.3 (99.1) | 310.8 (82.3) |  |  |
|  | Final | 324.3 (4.64) | 324.7 (4.62) | 340.5 (4.64) | 0.019 | 16.2 (3.30, 29.1) |
| Na – mg/d | Baseline | 3605 (948.4) | 3278 (895.4) | 3262 (926.0) |  |  |
|  | Final | 3234 (42.9) | 3296 (42.6) | 3417 (42.7) | 0.009 | 182.6 (63.4, 301.8) |
| K – mg/d | Baseline | 4387 (1022) | 4385 (999.6) | 4407 (1089) |  |  |
|  | Final | 4743 (56.7) | 4582 (56.7) | 4568 (56.8) | 0.053 | -175.0 (-332.3, -17.6) |

SFA denotes Saturated fatty acids. MUFA Monounsaturated fatty acids. PUFA Polyunsaturated fatty acids. EPA Eicosapentaenoic acid. DHA Docosahexaenoic acid and FA Fatty acids. ^1^ Baseline values are observed means (SD). ^2^ Final values are baseline-adjusted (least-squares) means (SE) and comparison among groups done with ANCOVA analysis ^3^ANCOVA analysis.

**Supplementary Figures**

**Supplementary Figure S1.** Flowchart of study participants.

**
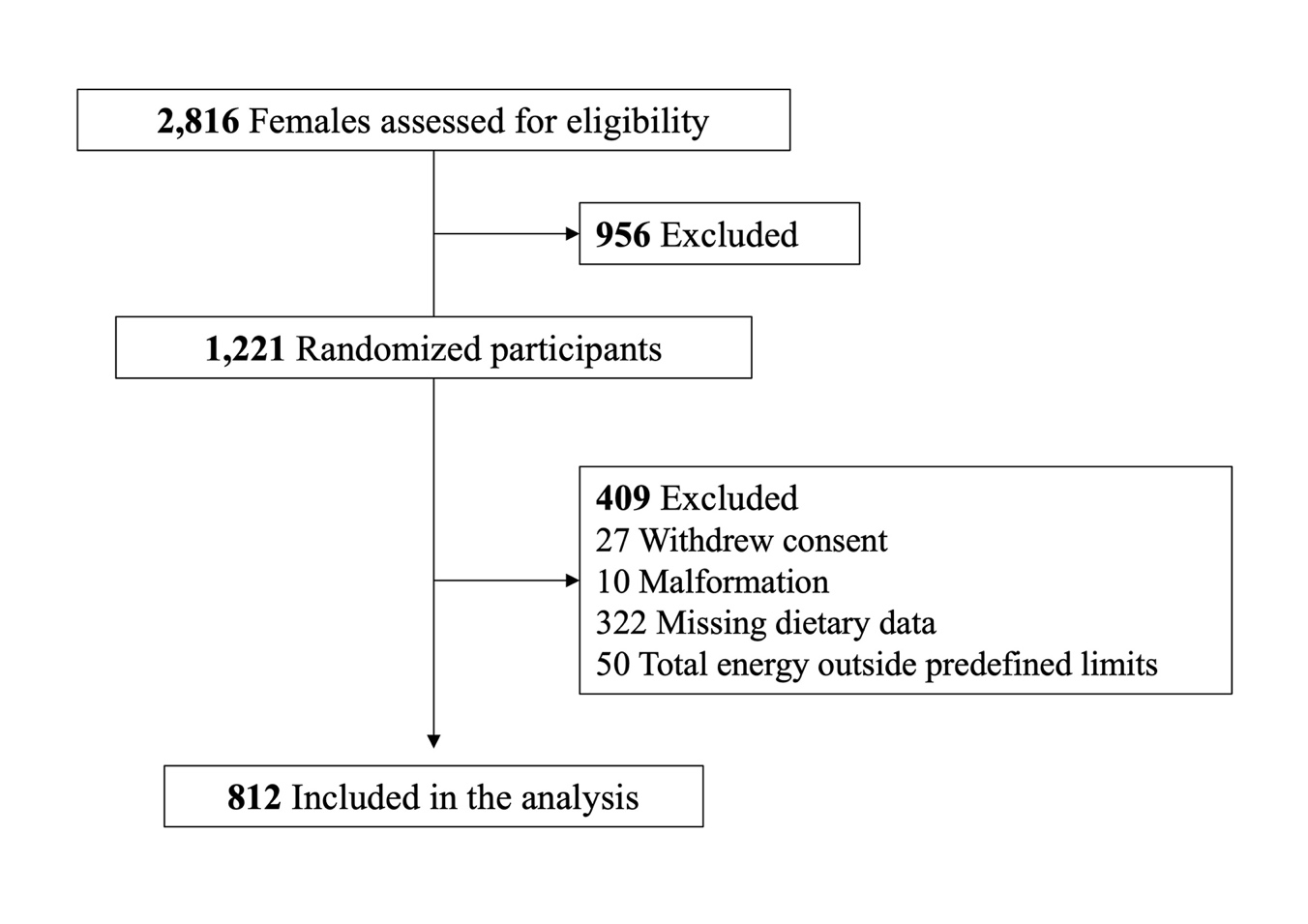
**
